# Characterization of sarcoidosis at a combined rheumatology-pulmonary clinic

**DOI:** 10.3389/fmed.2025.1646747

**Published:** 2025-09-15

**Authors:** Megan Schermerhorn, Daniel Seifer, Julianna Desmarais

**Affiliations:** ^1^Internal Medicine, Providence Portland Medical Center, Portland, OR, United States; ^2^Division of Pulmonary and Critical Care Medicine, Oregon Health and Science University, Portland, OR, United States; ^3^Division of Arthritis and Rheumatic Diseases, Oregon Health and Science University, Portland, OR, United States

**Keywords:** sarcoidosis, ILD, rheumatology, pulmonology, multidisciplinary

## Abstract

**Objective:**

The treatment landscape for sarcoidosis remains poorly defined, as there is no standardized approach to management. This study aims to characterize a cohort of sarcoidosis patients in a combined rheumatology-pulmonary clinic with regard to organ involvement and medications used. To our knowledge, this will be the first published manuscript to detail such a population, providing critical insight to inform and improve the care of patients with sarcoidosis in the multidisciplinary setting.

**Methods:**

This study was a retrospective chart review of sarcoidosis patients seen at the collaborative rheumatology-pulmonology clinic at Oregon Health & Science University during the first 5 years following the clinic’s inception (October 2018 to October 2023). Using ICD-10 diagnostic codes, 219 patient charts were identified. Of these, 37 were excluded for not meeting inclusion criteria, resulting in 182 charts eligible for data analysis. We used Excel spreadsheets to analyze our data.

**Results:**

119 (65.3%) of patients in our data base had multi-organ involvement of sarcoidosis. Treatment patterns differed between patients with multi-organ sarcoidosis and those with single-organ or isolated lung/lymph node involvement. Notable differences (in prescribing) were observed among patients with cardiac and neurologic manifestations. Additionally, patients with cardiac involvement demonstrated distinct demographic characteristics, including differences in age and SRY carrier status, when compared to the broader cohort.

**Conclusion:**

Our data highlights the complexity and heterogeneity of sarcoidosis seen in a combined rheumatology-pulmonary clinic. Further research is required to prospectively evaluate various pharmacologic treatment in these patients.

## Introduction

Sarcoidosis is a dysfunction of the immune system of uncertain etiology, characterized by the formation of small clusters of inflammatory cells that coalesce into granulomas throughout the body. Although commonly affecting the lungs and lymph nodes, it can manifest in nearly any organ and thus cause a wide spectrum of symptoms of highly variable severity from a nuisance (e.g., slow-growing adenopathy) to deadly (e.g., high-degree heart block). Given how much is unknown about this spectrum of dysfunction, sarcoidosis lacks a standardized treatment approach (1, 2). To advance the understanding of this condition and the care of affected patients, there exists an active call for collaboration between researchers globally to better characterize the disease (3, 4).

At the quaternary academic center Oregon Health and Science University (OHSU), a rheumatology-pulmonology collaborative clinic was founded in 2018 in an effort to speed communication and improve decision making in diagnosis and treatment. Each patient is seen by a dedicated pulmonologist and rheumatologist, with additional dedicated subspecialty providers involved with patients on the basis of known organ/system involvement (e.g., each patient with cardiac involvement follows with an OHSU Cardiologist who specializes in cardiac sarcoid, etc.). While other university centers report benefits from involving rheumatologists earlier in care for patients with interstitial lung damage (ILD) from sarcoidosis, there is a scarcity of reports detailing wholistically collaborative approaches to sarcoidosis for each patient such as practiced at OHSU (5).

This retrospective chart review aims to characterize the cohort of sarcoidosis patients in OHSU’s combined rheumatology-pulmonary clinic and will be the first published manuscript to detail such a population. Such characterization is vital to improve the general knowledge base surrounding effective approaches to the care of these complex patients. To that end, this manuscript descriptively reports the defining characteristics of the patient population and their current treatment regimens. Through showcasing this data, it supports the utility of a collaborative approach to this highly variable and complicated condition.

## Methods

This work presents a retrospective chart review of sarcoidosis patients attending the collaborative rheumatology-pulmonology clinic at a single quaternary academic medical center, Oregon Health and Sciences University (OHSU), during the first 5 years after clinic inception (from 10/2018 to 10/2023). By using ICD10 diagnosis codes, 219 charts were identified, of which 37 were excluded. The inclusion criteria were having a diagnosis of sarcoidosis (either clinical or biopsy-proven) with age over 18, seen between 10/1/2018 and 10/31/2023 for at least one outpatient visit with the pulmonary and/or rheumatology providers staffing the clinic. Patients without a diagnosis of sarcoidosis (6) or those not seen at OHSU’s collaborative clinic (25) were excluded. This project obtained authorization for chart review through the OHSU IRB. Data analyzed in Microsoft Excel and Mann–Whitney U tests and Chi-Squared tests were used to analyze for statistical significance.

## Results

There were 182 patients eligible for data review, of whom 81.8% of sarcoidosis cases were biopsy-proven. For the purposes of this manuscript, patients listed as male were presumably carriers of sex-determining region Y (SRY), and patients listed as female were presumably without SRY. Patient gender identity was obtained through the electronic health record review. The mean age of patients was 52.5 years old, of which 101 were female (55.4%) and 81 were male (44.5%). The patients had multiorgan involvement in 65.3% of cases (119/182). Total distribution of organ involvement for all patients is documented in Table 1.

**Table 1:** In all 182 charts reviewed, which organs were involved by number and percent.

Organ involved	Total number of patients with specified organ involvement (*n* = 182)
Pulmonary	146 (80.2%)
Cardiac	44 (24.1%)
Lymph	117 (64.2%)
Cutaneous	27 (14.8%)
Neurologic	17 (9.3%)
Joint	39 (21.4%)
Bone	14 (7.6%)
Spleen	21 (11.5%)
Liver	21 (11.5%)
Ocular	28 (15.3%)
Kidney	3 (1.6%)
Other (GI tract, GU tract, glandular, larynx, sinus, vascular, and/or vocal cords)	18 (9.8%)

There were treatment differences between patients who had sarcoidosis involvement of multiple organs compared to those with single-organ involvement or lung/lymph involvement, the latter two of which were combined into one comparison group. Furthermore, notable differences in treatment were observed among patients with cardiac and neurological manifestations. Additionally, there were notable demographic differences including age and SRY carrier status in patients with cardiac sarcoidosis when compared to the overall group. These differences are discussed further below and outlined in Tables 2–4. Of note, some patients in this cohort received combination therapy of Methotrexate and TNF-alpha inhibitors.

**Table 2:** Sarcoidosis involvement (multiple organs, single organ, cardiac, and neurologic) and the medication used for sarcoidosis treatment.

Medication	Multiorgan (*n* = 119)	Single organ (*n* = 63)	Cardiac (*n* = 44)	Neurologic (*n* = 17)
Corticosteroids	93 (78.1%)	43 (68.2%)	33 (75%)	14 (82.3%)
Methotrexate	84 (70.5%)	28 (44.4%)	32 (72.7%)	13 (76.4%)
Azathioprine	26 (21.8%)	8 (12.6%)	8 (18.1%)	4 (23.5%)
Hydroxychloroquine	26 (21.8%)	2 (3.1%)	3 (6.8%)	1 (5.8%)
Leflunomide	13 (10.9%)	3 (4.7%)	5 (11.3%)	2 (11.7%)
Mycophenolate	17 (14.2%)	2 (3.1%)	4 (9%)	6 (35.2%)
Sulfasalazine	3 (2.5%)	0	0	0
Cyclophosphamide	1 (0.8%)	0	0	1 (5.8%)
Anti-TNF biologics	43 (36.1%)	13 (20.6%)	18 (40.9%)	13 (76.4%)
JAK inhibitors	5 (4.2%)	1 (1.5%)	0	1 (5.8%)
Rituximab	1 (0.8%)	0	0	0
IL-6 inhibitors	3 (2.5%)	0	0	2 (11.7%) *
Cyclosporine	2 (1.6%)	0	0	1

*See Supplementary material A.

**Table 3:** Sarcoidosis involvement (multiple organs, single organ, cardiac, and neurologic) and patient identified gender distribution.

Gender patient identifies as	All charts reviewed (182)	Cardiac (*n* = 44)	Neurologic (*n* = 17)
Male	81 (44.5%)	26 (59%)	8 (47%)
Female	101 (55.5%)	18 (40.9%)	9 (52.9%)

**Table 4:** Sarcoidosis involvement (multiple organs, single organ, cardiac, and neurologic) and average age of first presentation to multidisciplinary clinic in years.

	All charts reviewed (182)	Cardiac (*n* = 44)	Neurologic (*n* = 17)
Average age of first presentation to clinic	52.4	57	47.15

### Multiorgan involvement

Multiorgan sarcoidosis involvement was present in 119/182 patients. The mean age of these patients was 52.7 years old, 68 (57.1%) of whom were female. The distribution of organs involved for these patients with multiorgan involvement are outlined in Table 5. The nonfractional mean number of systems involved in multiorgan involvement was 3. The medications used for the treatment for patients with multiorgan involvement are outlined in Table 2.

**Table 5:** In patients with multiorgan involvement, which organs were involved by number and percent.

Organ involved	Total number of patients with specified organ involvement (*n* = 119)
Pulmonary	97 (81.5%)
Cardiac	37 (31%)
Lymph	82 (68.9%)
Cutaneous	25 (21%)
Neurologic	16 (13.4%)
Joint	40 (33.6%)
Bone	14 (11.7%)
Spleen	21 (17.6%)
Liver	21 (17.6%)
Ocular	28 (23.5%)
Kidney	3 (2.5%)
Other (GI tract, GU tract, glandular, larynx, sinus, vascular, and/or vocal cords)	18 (15.1%)

### Single organ involvement

Single organ involvement or involvement isolated to lung and lymphatic systems was found in 63/182 patients with an average age of 54 years old, 33 (52.3%) of whom were female. The distribution of organs involved for patients with single organ involvement, or involvement isolated to lung and lymphatic systems, are outlined in Table 6. The medications used for treatment for patients with single organ involvement, or involvement isolated to lung and lymphatic systems, are outlined in Table 2. This category of single organ involvement also encompassed patients with isolated cardiac disease.

**Table 6:** In patients with single organ involvement, or organ involvement isolated to lung and lymph, which organs were involved by number and percent.

Organ involved	Total number of patients with specified organ involvement (*n* = 63)
Pulmonary	49 (77.7%)
Cardiac	7 (11.1%)
Lymph	35 (55.5%)
Cutaneous	2 (3.1%)
Neurologic	1 (1.5%)

### Cardiac sarcoidosis

Cardiac sarcoidosis was present in 44/182 patients (24.1%), 75.0% (33/44) of whom had biopsy-proven sarcoidosis. There was isolated cardiac involvement (i.e., no known other systems involved) in 7 cases (15.9%). Most (42/44) had undergone PET (either full body or cardiac PET) and 37/44 had undergone cardiac MRI. The average age of patients with cardiac sarcoidosis on presentation to the collaborative clinic was 57 years old, which included 26 males (59.0%) and 18 (40.9%) females. A Mann–Whitney U test found statistical significance when comparing the mean age of patients with cardiac sarcoidosis to the overall group, supporting that patient with cardiac sarcoidosis on average tended to be older (*p*-value 0.037). Chi-squared testing was performed to compare presumed SRY carrier status of cardiac sarcoidosis patients to the overall group, which showed statistical significance (*p*-value of 0.025).

Of the 44 patients with cardiac sarcoidosis, 11/44 presented with atrioventricular (AV) block, 11/44 with heart failure reduced ejection fracture (HFrEF), 9/44 with other underlying arrythmias other than ventricular tachycardia (VT) (primarily atrial fibrillation), 4/44 with cardiac arrest, 4/44 with recurrent VT without cardiac arrest, 1/44 with heart failure preserved ejection fraction (HFpEF), and 1/44 with hypertrophic cardiomyopathy. Few patients (3/44) were incidentally found to have cardiac involvement through PET/MRI imaging after first being diagnosed with sarcoidosis through a different organ system though did not have chart review of cardiac complications. The distribution of medications for cardiac sarcoidosis are outlined in Table 2. Many patients required multiple medications to treat cardiac sarcoidosis. In summary, 34/44 patients (77.2%) required two or more medications, and of that group 23/44 (52.2%) required three or more medications.

We individually reviewed treatments for patients who had presented with cardiac arrest to see whether these patients were treated differently than others with more mild phenotypes. All 4 patients who presented with cardiac arrest were started on corticosteroids. One patient was tapered off steroids without additional medications needed. The 3 remaining patients were started on methotrexate (MTX) as their initial non-steroidal immunomodulatory agent with one patient eventually being switched to azathioprine (AZA) due to side effects from MTX. Of note, all 3 patients were eventually switched to adalimumab due to ongoing cardiac sarcoid activity despite these initial treatments with antimetabolites (e.g., MTX, AZA, etc.).

### Neurological sarcoidosis

Neurologic sarcoidosis was diagnosed in 17/182 patients (9.3%), with the average age of presentation to clinic of 47.15 years. The SRY-carrier breakdown was eight males (47.0%) and 9 females (53%). 70.5% (6) of the neurologic sarcoidosis cases were biopsy-proven. When comparing patients with neurologic sarcoidosis to the overall group regarding both age and SRY-carrier status, there were no statistically significant differences of note. There was isolated neurologic involvement in 1 patient, who did not have biopsy-proven disease and was treated with AZA, steroids, and an anti-TNF agent (infliximab). Central nervous system (CNS) involvement was seen in 15/17 patients while 2/17 had peripheral nervous system (PNS) involvement. Of the 15 patients with CNS involvement, presentations included leptomeningeal enhancement, white matter lesions, lesions within the nerve root, transverse myelitis (3/17 patients), and cranial nerve involvement such as optic neuritis. Of the patients with PNS involvement, presentations included small fiber neuropathy and abnormalities on electromyography (EMG).

For the 17 cases of neurological sarcoidosis, treatment is outlined in Table 2. Of the 13 patients who were prescribed anti-TNF inhibitors, some transitioned between various options in search of clinical efficacy. The anti-TNF inhibitors that were used included adalimumab (7), infliximab (8), etanercept (1), and certolizumab pegol (1). Similar to patients with cardiac sarcoidosis, several patients with neurologic sarcoidosis required treatment with multiple drug categories with a minimum of two and a maximum of six medications from different classes. Most (15/17, 88.2%) patients were treated with 2 + medication classes, and 70.5% (12/17) were treated with 3 + medication classes.

## Discussion

In conclusion, our data highlights the complexity and heterogeneity of patients with sarcoidosis. Multiorgan sarcoidosis was present in 64.6% of patients, a number notably higher than seen in other reports (1). This data suggests that, as a referral center, OHSU is more likely to encounter complex manifestations of sarcoidosis, as patients with less severe disease are less often referred to this type of setting. These patients’ treatment regimens were similarly complex. Corticosteroids, MTX, and AZA were the most common DMARDs used for treatment, while anti-TNF inhibitors (distinctly adalimumab and infliximab) were the most common among targeted molecules and monoclonal antibodies. The use of infliximab and adalimumab aligns with existing literature on treating refractory sarcoidosis (9, 10).

Published literature on suggested treatment regimens for sarcoidosis is generally limited to observational studies. As such, the application of those publications toward treatment for patients with multi-organ sarcoidosis is problematic. The usual observed standard treatment for sarcoidosis includes steroids with MTX and AZA as first line steroid-sparing agents; anti-TNF inhibitors are used for patients with contraindications to these treatments or with severe or refractory disease (1, 2, 11). Our observed trends highlighted stark differences, particularly between patients with multi-organ involvement compared to single organ (or limited to lung and lymph nodes) as well as variations between treatment of cardiac and neurologic involvement. Targeted molecules or monoclonal antibodies were used to treat 36.1% of patients with multiorgan sarcoidosis compared to 20.6% of patients with single organ involvement or involvement isolated to lung and lymphatic systems. Using Chi-squared testing, this was found to be statistically significant (*p*-value 0.031). The statistically significant difference in medication management further supports early involvement of the multi-disciplinary team for patients with multi-organ sarcoidosis.

Cardiac sarcoidosis deserves specific focus given the complications involved (e.g., ventricular arrythmias, heart block, heart failure, sudden cardiac death, etc.) (11). Though involvement is rare, the severity and sudden nature of these outcomes warrants careful surveillance of all patients with sarcoidosis. OHSU’s collaborative clinic approach addresses basic screening with yearly electrocardiogram (ECG) and approaches all reports of abnormalities with aggressive workup (e.g., event monitor, echocardiogram, combined cardiac MRI/PET, etc.). These tests are interpreted with direct assistance from the cardiologist specializing in cardiac sarcoidosis that participates in the collaborative clinic. Once cardiac involvement moves from speculative to suspected or confirmed, affected patients are formally followed by this same cardiologist. The collaborative approach allows for not only dedicated providers but for dedicated weekly time for case discussion, allowing for more rapid diagnosis and nuanced treatment approaches. This is especially relevant for patients with sarcoidosis confined to the heart, given the challenges in interpreting combined PET/MRI results and the low negative predictive value (NPV) associated with the relatively risky endomyocardial biopsy (12). Most patients in our study who were diagnosed with cardiac sarcoidosis underwent PET, MRI, both separate scans, or a combined scan (42/44 and 37/44 respectively).

Corticosteroids are typically accepted as first line treatment, but there are no standardized recommendations regarding duration and dosage and these reviews are again limited to retrospective cohort and case series (7, 11, 13). Longitudinal treatment regimens are even less clear. There are case series evaluating anti-TNF alpha inhibitors, including one retrospective single-center trial evaluating adalimumab which showed benefit in suppressing cardiac inflammation as measured through F-FDG uptake in PET/CT (8). In our clinic, 40.9% of patients with cardiac sarcoid involvement were treated with targeted molecule or monoclonal antibody at some point in their treatment course, and 77.2% (34) of patients required two or more medications.

Available literature and knowledge surrounding neurologic sarcoidosis is very limited. Neurologic sarcoidosis can involve both the peripheral and central nervous systems and can present with a variety of symptoms including headache, nausea, ataxia, changes in vision, and even seizures (14). Treatment similarly generally involves corticosteroids, with multiple steroid-sparing agents having been used including MTX, AZA, and hydroxychloroquine (HCQ) (6, 14). Anti-TNF therapy has demonstrated efficacy in several case reports and small case series particularly in refractory cases (6, 9, 10, 14, 15). In our review, 76.4% of patients with neurologic sarcoidosis involvement were treated with targeted molecule or monoclonal antibody at some point in their treatment courses. Additionally, of the patients with neurologic sarcoidosis, 88.2% were treated with 2 + medication classes and 70.5% were treated with 3 + medication classes. The fact that multiple drugs are required in the majority of neurologic sarcoidosis cases supports other existing reports published indicating that neurologic sarcoidosis is often refractory to treatment (6). The overall variability in treatment regimens we found supports a need for further research aimed at identifying preferential, unified approaches to pharmacologic therapy.

Regarding the composition of care teams for these complex patients, our data supports a multi-subspecialty collaborative approach including—at minimum—rheumatologists and pulmonologists. For patients with multiple organ involvement or those at risk for refractory disease, consultation with other specialists is strongly recommended. Early involvement of appropriate experts is especially crucial for individuals with neurologic and cardiac involvement.

Our data report presented here has substantial limitations shared with much of the published literature on complex sarcoidosis care: it is retrospective, lacks well-characterized standardized disease activity outcomes, and provides only limited insight into the choices of a given course of therapy apart from disease being refractory to initial choice(s). In order to advance the study of best practices in this area, we intend to move to prospective collection of disease activity measures in the future as well as making attempts at evaluating outcomes between varying treatment approaches. Additionally, further retrospective analysis to guide prospective work will be conducted using the data we have organized and presented in this manuscript.

## Conclusion

The data presented describe a multi-subspecialty collaborative approach to the care of complex sarcoidosis at a single quaternary academic center in the United States. The findings in this study are descriptive and highlight the complexity of the patient cohort and the treatment patterns observed. This descriptive data serves as a foundational step for future prospective research into the patients seen at collaborative ILD clinics in the academic setting.

## Data Availability

The raw data supporting the conclusions of this article will be made available by the authors, without undue reservation.
