# Supplementary material for: Characterization of sarcoidosis at a combined rheumatology-pulmonary clinic
Source: Front Med (Lausanne). 2025 Sep 15;12:1646747. doi: 10.3389/fmed.2025.1646747 (PMC12477196; doi:10.3389/fmed.2025.1646747)
Supplement: Supplementary file 1 [file Data_Sheet_1.docx]

**Supplemental Material**

1. Two patients with neurological sarcoidosis were prescribed tocilizumab. These charts were reviewed further: both patients were female; one patient was biopsy-proven, and the other was not; one patient had neurological sarcoidosis and eye involvement (2 systems), while the other with neurological, lung, joints, and lymph involvement (4 organ systems). Beyond tocilizumab, treatments used in these two patients included methotrexate (2), azathioprine (1), mycophenolate mofetil (1), corticosteroids (1), and anti-TNF (Adalimumab (1), Infliximab (1), etanercept (1), and certolizumab pegol (1). For both of these patients, tocilizumab was used after both first- and second-line treatments failed. No evidence for clinical response to tocilizumab was seen.
